# Sex Bias in Infectious Disease Epidemiology: Patterns and Processes

**DOI:** 10.1371/journal.pone.0062390

**Published:** 2013-04-24

**Authors:** Felipe Guerra-Silveira, Fernando Abad-Franch

**Affiliations:** 1 Instituto Leônidas e Maria Deane – Fiocruz Amazônia, Manaus, Amazonas, Brazil; 2 School of Medicine, Universidade do Estado do Amazonas, Manaus, Amazonas, Brazil; University of Hong Kong, Hong Kong

## Abstract

**Background:**

Infectious disease incidence is often male-biased. Two main hypotheses have been proposed to explain this observation. The physiological hypothesis (PH) emphasizes differences in sex hormones and genetic architecture, while the behavioral hypothesis (BH) stresses gender-related differences in exposure. Surprisingly, the population-level predictions of these hypotheses are yet to be thoroughly tested in humans.

**Methods and Findings:**

For ten major pathogens, we tested PH and BH predictions about incidence and exposure-prevalence patterns. Compulsory-notification records (Brazil, 2006–2009) were used to estimate age-stratified ♂:♀ incidence rate ratios for the general population and across selected sociological contrasts. Exposure-prevalence odds ratios were derived from 82 published surveys. We estimated summary effect-size measures using random-effects models; our analyses encompass ∼0.5 million cases of disease or exposure. We found that, after puberty, disease incidence is male-biased in cutaneous and visceral leishmaniasis, schistosomiasis, pulmonary tuberculosis, leptospirosis, meningococcal meningitis, and hepatitis A. Severe dengue is female-biased, and no clear pattern is evident for typhoid fever. In leprosy, milder tuberculoid forms are female-biased, whereas more severe lepromatous forms are male-biased. For most diseases, male bias emerges also during infancy, when behavior is unbiased but sex steroid levels transiently rise. Behavioral factors likely modulate male–female differences in some diseases (the leishmaniases, tuberculosis, leptospirosis, or schistosomiasis) and age classes; however, average exposure-prevalence is significantly sex-biased only for *Schistosoma* and *Leptospira*.

**Conclusions:**

Our results closely match some key PH predictions and contradict some crucial BH predictions, suggesting that gender-specific behavior plays an overall secondary role in generating sex bias. Physiological differences, including the crosstalk between sex hormones and immune effectors, thus emerge as the main candidate drivers of gender differences in infectious disease susceptibility.

## Introduction

Infectious diseases rarely affect males and females equally, despite even demographic sex ratios. This has been observed in humans [1]–[5] and other animals, from birds to invertebrates [6]–[10]. Two major, not mutually exclusive hypotheses have been put forward to explain such sex-biased patterns [3]. The physiological hypothesis (PH) posits that the interactions between sex hormones and the immune system render one sex more susceptible to infection and disease, with genetic (chromosome) differences likely playing also a role [3], [6], [8], [9], [11]–[17]. Since sex and immunity are both critical to fitness and energetically costly, immune effectors and sex hormones engage in an intense physiological crosstalk [6]–[18]; in mammals, sex steroid immunomodulation has been linked to higher infection rates in males, with testosterone down-regulating and estrogen promoting T-helper(Th)1- and antibody-dominated responses [3], [6]–[9], [16]. On the other hand, the behavioral hypothesis (BH) posits that sex-biased infection rates emerge from sex-specific exposure to contagion [3], [19]. Higher exposure usually arises from differences in behavior (foraging, combat), although sexual size dimorphism may also be important [8], [19]. In some human populations, gender-related behavioral differences may render one sex more exposed to certain pathogens [2]–[4], [6], [20], [21].

Several studies have addressed human male-female differences in overall mortality (e.g., [22]), susceptibility to allergic and autoimmune diseases (e.g., [17]), or individual infectious disease risk (e.g., [23]–[27]); yet, and surprisingly, a critical, comprehensive test of the major hypotheses outlined above is currently unavailable (but see ref. [3]). Aiming to address this gap, here we use a hypothesis-driven approach and two complementary, large datasets to investigate the roles of physiology and behavior as potential drivers of sex-biased infectious disease incidence in humans.

## Methods

We consider the following major infectious diseases: American cutaneous and visceral leishmaniasis, schistosomiasis, pulmonary tuberculosis, lepromatous and tuberculoid leprosy, typhoid fever, leptospirosis, meningococcal meningitis, hepatitis A, and severe dengue fever. For each, we test the key predictions of the PH and BH as outlined below (see also Table 1).

**Table 1:** Testing hypotheses on sex-biased infection rates: pathogen traits and main expectations on sex-biased incidence under the physiological hypothesis (PH) and the behavioral hypothesis (BH).

Disease*	Pathogen		Predictions			
			Infants		Adults	
	Taxonomy and classification	Transmission	PH	BH#	PH	BH
CL	*Leishmania* spp. (intracellular protozoa)	Vector-borne (forest sandflies)	♂>♀	♂ = ♀	♂>♀	♂>♀
VL	*Leishmania infantum* (intracellular protozoan)	Vector-borne (peridomestic sandflies)	♂>♀	♂ = ♀	♂>♀	♂>♀
SCH	*Schistosoma mansoni* (helminth)	Water-borne (through intact skin)	♂>♀	♂ = ♀	♂≥♀†	♂>♀
TB	*Mycobacterium tuberculosis* (intracellular mycobacterium)	Person-to-person	♂>♀	♂ = ♀	♂>♀	♂>♀
LL	*Mycobacterium leprae* (intracellular mycobacterium)	Person-to-person	♂>♀	♂ = ♀	♂>♀	♂ = ♀
TL	*Mycobacterium leprae* (intracellular mycobacterium)	Person-to-person	♀>♂	♂ = ♀	♀>♂‡	♂ = ♀
TF	*Salmonella* Typhi (intracellular bacterium)	Food-borne	♂>♀	♂ = ♀	♂>♀	♂ = ♀
LE	*Leptospira interrogans* (extracellular bacterium)	Water-borne (through skin wounds)	♂>♀	♂ = ♀	♂>♀	♂>♀
MM	*Neisseria meningitidis* (extracellular bacterium)	Person-to-person	♂>♀	♂ = ♀	♂>♀	♂≥♀
HA	Hepatitis A virus (RNA virus)	Food/water-borne	♂>♀	♂ = ♀	♂>♀	♂ = ♀
SDF	Dengue virus (RNA virus)	Vector-borne (urban mosquitoes)	♂ = ♀	♂ = ♀	♀>♂‡	♂ = ♀

* American cutaneous leishmaniasis (CL), American visceral leishmaniasis (VL), schistosomiasis (SCH), community-acquired pulmonary tuberculosis in HIV-negative subjects (TB), lepromatous leprosy (LL), tuberculoid leprosy (TL), typhoid fever (TF), leptospirosis (LE), meningococcal meningitis (MM), acute hepatitis A (HA), and severe dengue fever (SDF: dengue hemorrhagic fever and dengue shock syndrome).

# BH predictions for infants apply equally to children.

† The PH also predicts much higher male bias in exposure (♂>>♀) than disease (♂≥♀).

‡ The PH predicts this female bias to disappear among the elderly.

### Hypotheses and Predictions

Under the PH, infectious disease incidence is predicted to be similar among male and female children, who have similarly low levels of sex hormones; importantly, however, the transient rise of sex steroid levels in the first year of life, known as “minipuberty” [17], should yield patterns resembling more those seen after puberty than those seen in childhood. In age classes in which sex hormones are physiologically active (infancy, puberty, and reproductive period), the PH predicts that disease incidence should be male-biased for the leishmaniases, pulmonary tuberculosis, lepromatous leprosy, typhoid fever, leptospirosis, meningococcal meningitis, and hepatitis A; in contrast, female bias is predicted when strong immune responses (i) enhance pathogenesis (such as in severe dengue and Manson’s schistosomiasis) or (ii) favor a certain form of disease presentation (such as the milder, tuberculoid forms of leprosy, *vs.* the more severe, lepromatous forms, which are predicted to be male-biased). Finally, male-female differences are overall expected to decrease among the elderly, when estradiol levels sharply decrease in women but androgen levels remain high among men [17], [28]; more specifically, sex bias should (i) disappear for diseases in which female bias is expected to result from immune response-related pathogenesis, and (ii) decrease slightly to moderately for diseases in which male bias is expected to result from androgen down-regulation of immune responses.

On the other hand, the BH predicts that male-female differences should not appear in same-behavior age classes (mainly infancy) and should be overall absent for diseases whose transmission (typhoid fever, hepatitis A) or clinical progression (tuberculoid *vs.* lepromatous leprosy, meningococcal meningitis, severe dengue forms) do not depend upon host behavior. Male-biased patterns are expected for the leishmaniases, schistosomiasis, tuberculosis, and leptospirosis – particularly after puberty, when behavior-related exposure is usually higher among males. In addition, bias patterns are predicted to differ in populations with contrasting sociological-behavioral profiles, which can lead to different levels of gender-biased exposure. Thus, the risk of infection with vector-borne (*Leishmania*) and water-borne (*Leptospira*, *Schistosoma*) pathogens should be particularly male-biased in rural settings, where men engage more often in risky activities such as agriculture. Since the PH predicts similar outcomes for individuals of the same age class, regardless of sociological background, any difference in male bias between rural and urban populations would reflect, at least partially, behavioral risk factors.

Finally, the PH and the BH make contrasting predictions regarding indices of exposure to pathogens: they should be sex-unbiased under the PH, and sex-biased under the BH, for age classes whose members engage in gender-specific behavior (mainly after puberty). Such indices are provided by exposure-without-disease surveys that make use of antibody detection (via serology) and, in some instances, other measures of sensitization (e.g., delayed-type hypersensitivity skin tests) or direct evidence of infection in apparently healthy subjects (e.g., *S. mansoni* egg shedding in stools or nasopharyngeal *Neisseria meningitidis* carriage). The main predictions of each hypothesis are summarized in Table 1.

### The Data

Our *disease incidence* data consist of sex- and age-stratified compulsory-notification records published by the Brazilian Ministry of Health (http://dtr2004.saude.gov.br/sinanweb) for the 2006–2009 period. For general comparisons across age classes, we used countrywide data and the official population estimates for each age class, sex, and year provided by the Brazilian Institute for Geography and Statistics (IBGE) (http://www.ibge.gov.br) (Dataset S1). For some diseases, we selected cases with specific characteristics so that clear-cut predictions could be derived in each instance. Thus, for leprosy we considered only new, confirmed cases of the two polar forms of the disease – lepromatous and tuberculoid leprosy. For the analyses of tuberculosis, we considered only new, confirmed cases of community-acquired pulmonary disease in HIV-negative subjects. For hepatitis A, we selected only new, confirmed acute cases. For dengue, we selected only new, confirmed cases of the severe forms of the disease (i.e., dengue hemorrhagic fever and dengue shock syndrome). This latter choice follows from two facts: first, immunity to dengue virus depends on whether the case is a primary or a secondary infection, and this information is unavailable in the datasets we used; in contrast, severe forms of dengue are epidemiologically associated with secondary infections with a heterologous viral serotype, with antibody-dependent enhancement probably playing a key role in pathogenesis [29], [30]. Therefore, we were able to make clear-cut predictions for the severe forms of dengue, but not for dengue in general.

To compare sociologically distinct populations, we built rural/urban contrasts by selecting the Brazilian states with highest incidence (>200 new, confirmed, autochthonous cases notified in 2006) of cutaneous and visceral leishmaniasis, schistosomiasis, and leptospirosis, and used the detailed demographic data (age- and sex-stratified rural and urban population by municipality) available from the population count carried out in 2006–2007 by the IBGE. For cutaneous leishmaniasis, we separately analyzed data from the geographic areas where either *Leishmania guyanensis* or *Le. braziliensis* are the predominant etiological agents; see Dataset S2 for details on geographic coverage.

Our meta-analyses of published *exposure-prevalence* surveys are based on non-systematic article searches (Text S1) in PubMed, ISI Web of Knowledge, Scopus, and Google Scholar; query terms included the disease/pathogen name (e.g., “leishman*”) and different combinations of keywords – mainly “prevalence”, “seroprevalence”, “serology”, and “population-based”. No time limits were set. To keep results maximally comparable with our incidence data, we prioritized Brazilian studies, but surveys carried out elsewhere were also considered. We screened titles and abstracts to select population-based exposure-prevalence surveys in which the results for males and females were reported separately (the only inclusion/exclusion criterion), and noted the numbers of tested and positive subjects, the outcome measure, the diagnostic methods used, the study setting, and the age range of the subjects. Considering Brazilian official incidence records and our complementary appraisal of the literature on exposure-prevalence, our analyses made use of data from about 497,000 individual disease cases or diagnostic test results. We note that, in a few studies, individual subjects were tested with more than a single procedure, and emphasize that, even if document searches were non-systematic, none of the retrieved studies was excluded because it did or did not reveal sex biases – only those not reporting sex-specific results were.

### Data Analysis

To assess male-female differences in *disease incidence*, we estimated male:female incidence rate ratios (IRRs) and their 95% confidence intervals (CIs); for each disease, year, and age class, the ♂:♀ IRR is given by IRR = (*C*♂/*N*♂)/(*C*♀/*N*♀), where *C* is the number of disease cases notified to the Brazilian Ministry of Health and *N* is the population estimate provided by the IBGE. The variance of the log_e_IRR was estimated as Var(log_e_IRR) = 1/*C*♂ +1/*C*♀ − 1/*N*♂ − 1/*N*♀. IRRs for each disease, year, and age class were then combined through a meta-analytic approach using DerSimonian-Laird random-effects models, with inverse-variance weighting and α set to 0.05 [31], [32]. In a few cases, we used cumulative incidence (total number of cases during a given time-period divided by the population at the start of that period) to derive male:female IRRs (see also Text S2).

For the assessment of *exposure-prevalence* in apparently healthy subjects, we used the data from each of the published reports we retrieved to estimate the male:female odds ratio (OR) and its 95%CI; the ♂:♀ OR is given by OR = [*C*♂/(*N*♂−*C*♂)]/[*C*♀/(*N*♀−*C*♀)], with Var(log_e_OR) = 1/*C*♂ +1/(*N*♂−*C*♂) +1/*C*♀ +1/(*N*♀−*C*♀); here, *C* is the number of subjects classified as infected/positive and *N* is the number of subjects tested. These study-specific ORs were also summarized using DerSimonian-Laird random-effects models with inverse-variance weighting and α = 0.05 [31], [32]. The analyses described thus far were conducted using Review Manager 5.1 (The Cochrane Collaboration). We intentionally avoided formal null hypothesis significance testing [33]; IRR/OR 95%CIs including 1 indicate that male-female differences are not statistically significant at the 5% level. To compare simple proportions, we estimated their 95%CI limits using the Agresti-Coull method [34]. Sensitivity analyses examining the consistency of results across years and their robustness to incomplete reporting and the choice of analytical procedures are provided in Text S2.

## Results

**Figure 1:**
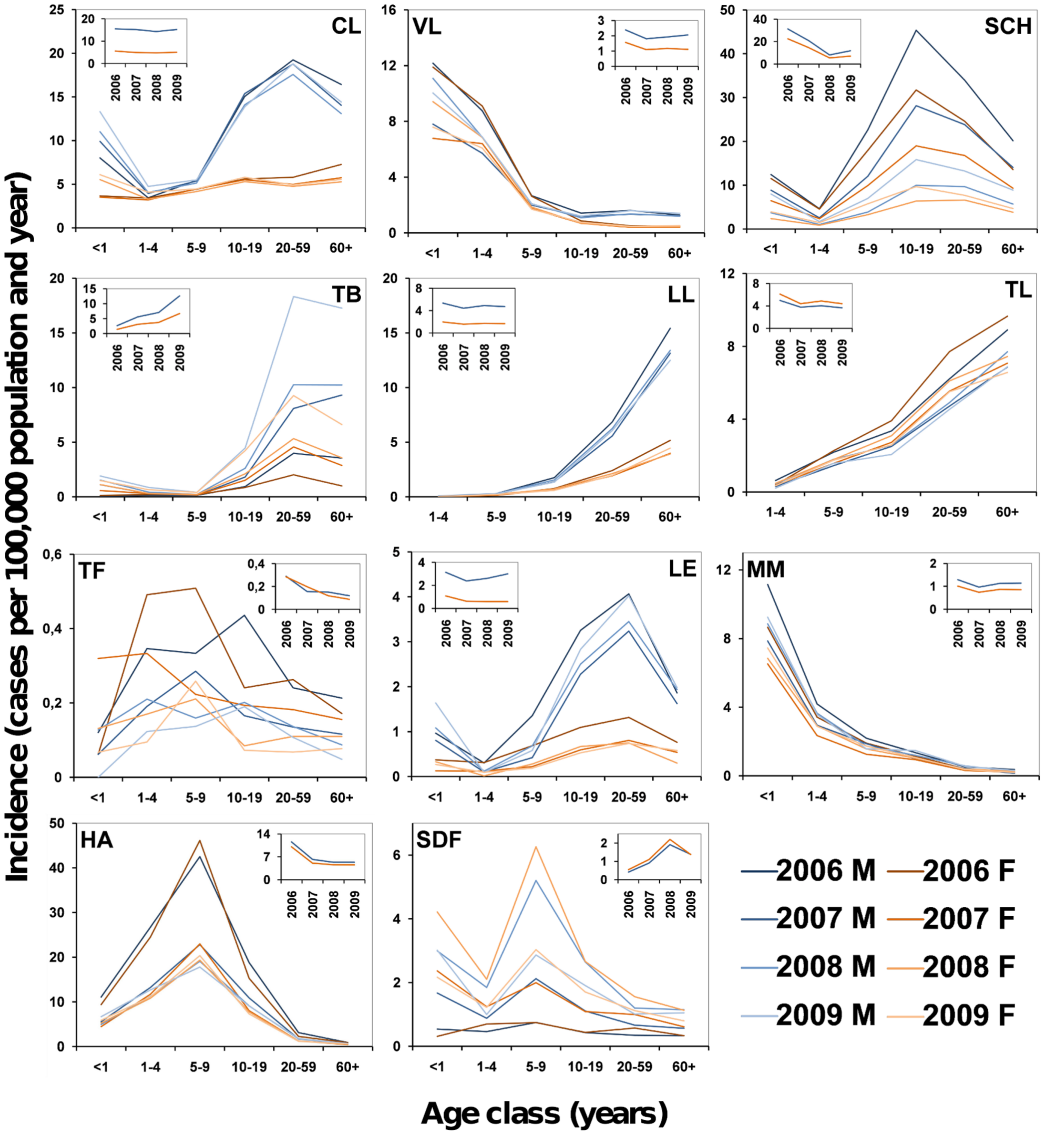
Infectious disease incidence in Brazil: sex- and age class-stratified incidence profiles (cases/100,000 population). Diseases: American cutaneous (**CL**) and visceral leishmaniasis (**VL**); schistosomiasis (**SCH**); pulmonary tuberculosis (**TB**); lepromatous leprosy (**LL**); tuberculoid leprosy (**TL**); typhoid fever (**TF**); leptospirosis (**LE**); meningococcal meningitis (**MM**); hepatitis A (**HA**); and severe dengue fever (**SDF**). Incidence (2006–2009) was computed from Brazilian compulsory-notification records and official demographic data for males (**M**, blue lines) and females (**F**, orange-red lines). Age classes (in years) are given on the *x* axes. Insets present overall annual incidence for 2006–2009 (blue, males; orange, females). See main text and Dataset S1 for details.

Crude incidence data (Figure 1) revealed patent post-pubertal male bias in cutaneous leishmaniasis, tuberculosis, lepromatous leprosy, and leptospirosis. More subtle differences appeared also in visceral leishmaniasis, schistosomiasis, and meningococcal meningitis, whereas no clear sex-specific pattern seemed to emerge from hepatitis A and typhoid fever data. Incidence appeared to be slightly female-biased after puberty for tuberculoid leprosy, and overall for severe dengue fever. Figure 1 also suggests that male bias is larger among infants than across childhood for cutaneous and visceral leishmaniasis, schistosomiasis, meningococcal meningitis, and leptospirosis. Incidence patterns were remarkably consistent across years except for typhoid fever, the least common among the diseases we studied; data on laboratory confirmation of hepatitis A cases were unavailable for 2006, which inflates incidence in both sexes. While overall suggestive of support for the PH, these data do not allow for a detailed quantitative appraisal of sex effect-sizes, which we therefore estimated using random-effects models; below we present detailed results by disease.

### Cutaneous Leishmaniasis

**Figure 2:**
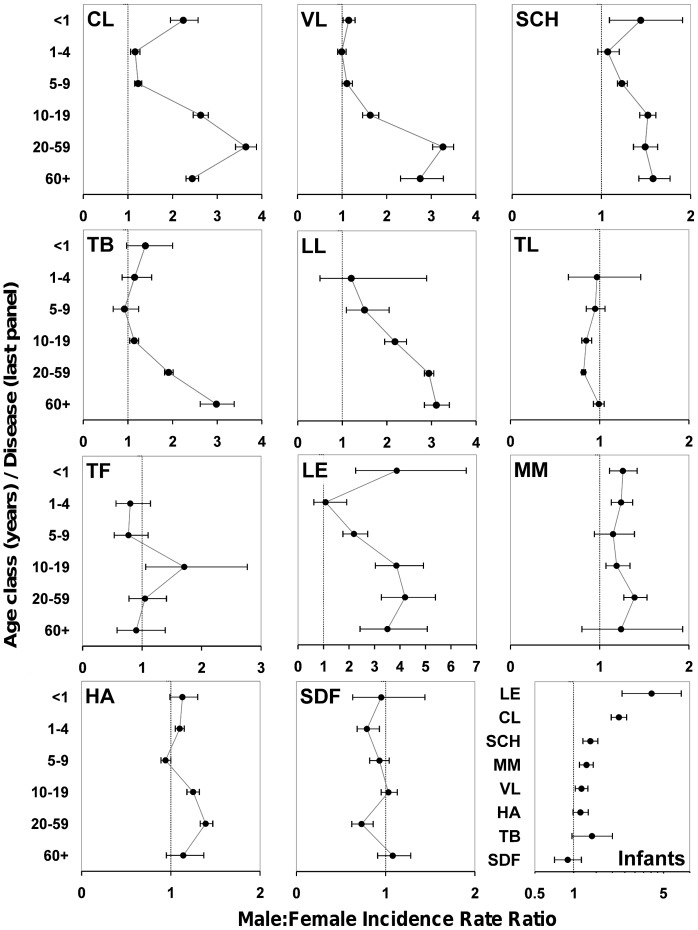
Infectious disease incidence in Brazil: male:female incidence rate ratios (IRRs) and 95% confidence intervals (CIs) computed from compulsory-notification records and official demographic data. Diseases: American cutaneous (**CL**) and visceral leishmaniasis (**VL**); schistosomiasis (**SCH**); pulmonary tuberculosis (**TB**); lepromatous leprosy (**LL**); tuberculoid leprosy (**TL**); typhoid fever (**TF**); leptospirosis (**LE**); meningococcal meningitis (**MM**); hepatitis A (**HA**); and severe dengue fever (**SDF**). Circles are random-effects point estimates computed from Brazilian compulsory-notification annual incidence records (2006–2009). IRR >1 indicates male-biased incidence; the vertical line at IRR = 1 indicates no sex bias. When CIs include 1, sex bias is not statistically significant at the 5% level. Age classes are given on the *y* axes; a few IRRs could not be estimated due to small numbers of incident cases. The last Panel (labeled ‘**Infants**’) compares cumulative incidence (2006–2009) among infants (<1 year old); despite the likely absence of sex-related behavior/exposure differences, significant male bias is seen in several diseases. See main text and Dataset S1 for details.

**Figure 3:**
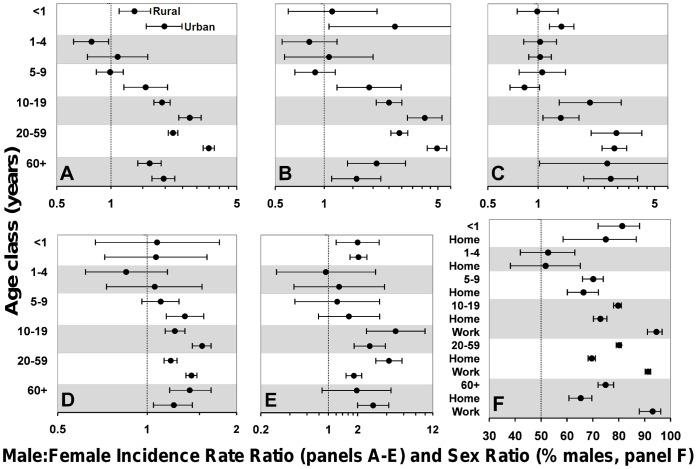
Infectious disease incidence in Brazil: sociological contrasts. Panels **A-E**: rural and urban male:female incidence rate ratios (IRRs) and 95% confidence intervals (CIs) for American cutaneous leishmaniasis in Brazilian sub-regions where *Leishmania braziliensis* (Panel **A**) or *Le. guyanensis* (Panel **B**) are the primary etiological agent; American visceral leishmaniasis (Panel **C**); schistosomiasis (Panel **D**); and leptospirosis (Panel **E**). Each age class (*y* axes) is represented by a gray or white band, with rural and urban estimates given as the upper and lower value within each band (as illustrated for infants under 1 year of age in Panel **A**); IRR >1 indicates male-biased incidence; the vertical line at IRR = 1 indicates no sex bias; when CIs include 1, sex bias is not statistically significant at the 5% level. Panel **F**: percentage of males (with 95%CIs) among 9498 incident leptospirosis cases (Brazil, 2006–2010); for each age class (grey/white band), the overall value is followed by infection site-specific estimates for the home (all age classes) and work environments (age classes >10); the vertical line at 50% indicates even demographic sex ratios. IRRs and CIs were computed from compulsory-notification records and official demographic data. See main text and Dataset S2 for details.

**Figure 4:**
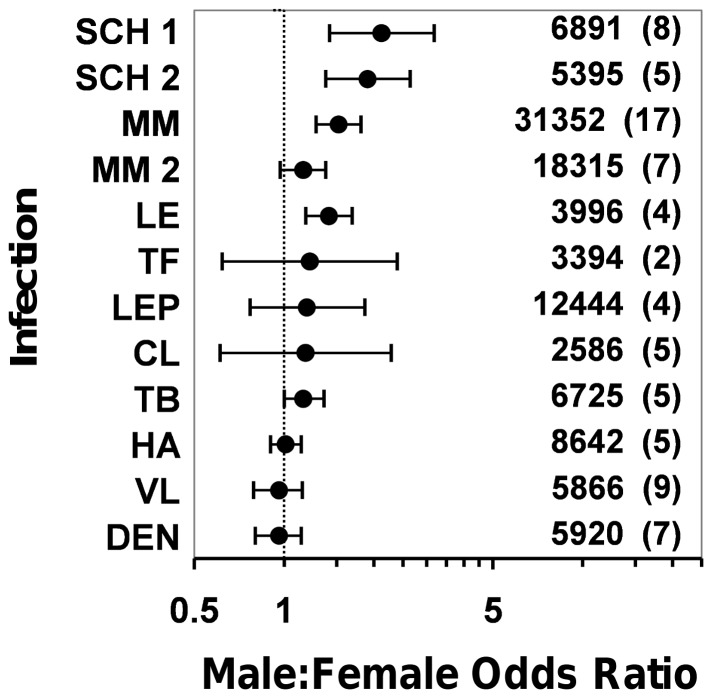
Sex bias in exposure to human pathogens: published exposure-without-disease surveys. The data reveal no sex bias for *Leishmania* spp. (**CL**, cutaneous forms; **VL**, visceral forms); *Mycobacterium leprae* (**LEP**); *Salmonella enterica* serovar Typhi (**TF**); Hepatitis A virus (**HA**); Dengue virus (**DEN**); or *Mycobacterium tuberculosis* (**TB**). Exposure to *Schistosoma mansoni* (**SCH 1**, as determined by the Kato-Katz technique; **SCH 2**, as determined by immunological tests); and *Leptospira interrogans* (**LE**) is male-biased. *Neisseria meningitidis* (**MM**) is more often carried by adult men, likely because of male-biased risk factors such as smoking; **MM 2** is a subset analysis of surveys involving children or high-school students, which reveals no sex bias; furthermore, many papers reporting a “non-significant” gender difference do not present the actual figures. Estimates (*x* axis) are random-effects odds ratios with 95% confidence intervals; when these include 1, sex bias is not statistically significant at the 5% level. The numbers of individual tests and published studies (in parentheses) analyzed are also given to the right of each estimate.

Male infants are twice as likely to develop clinical cutaneous leishmaniasis as females (Figure 2, Table 2); this bias was observed in all years from 2006 to 2010 (Text S2), and was present in both rural and urban settings except for rural areas north of the Amazon river (Figure 3B). Infant male bias shrinks by ∼48% in early and by ∼45% in late childhood. With the onset of puberty, the magnitude of male bias reaches values slightly larger (∼17%) than those of infancy. It then soars to a peak IRR value >3.5 in the reproductive-age population, and declines back to puberty/infancy values among the elderly (Figure 2, Table 2). Male:female IRR values are higher in urban than in rural settings in late childhood, puberty, and adulthood. This was consistently observed in areas where *Le. guyanensis* is the dominant agent of cutaneous leishmaniasis and in areas where *Le. braziliensis* predominates, but 95%CIs were much wider for *Le. guyanensis* due to the smaller number of records (Figure 3A, 3B). Published prevalence survey data suggest that exposure to the parasites causing cutaneous leishmaniasis is sex-unbiased (Figure 4). Surveys based on both leishmanin skin tests and antibodies yield consistent results: in Brazil (random-effects OR 1.18, 95%CI 0.61–2.28; Figure 4) [35]–[39] and elsewhere (e.g., ref. [40]: OR 1.20, 95%CI 0.94–1.53), male and female subjects are at similar risk of exposure (overall random-effects OR 0.99, 95%CI 0.72–1.36, *N* = 3566 tests).

**Table 2:** Sex bias in infectious disease epidemiology: age-stratified male:female incidence rate ratios and 95% confidence intervals computed from Brazilian compulsory-notification records and official demographic data (see Dataset S1).

Disease	Mean (random-effects) male:female incidence rate ratio, 2006–2009 (95% confidence interval)					
	Infancy (<1)	Early childhood (1–4)	Late childhood (5–9)	Puberty (10–19)	Reproductive (20–59)	Elderly (>60)
Cutaneous leishmaniasis	**2.24 (1.95–2.57)**	**1.16 (1.06–1.27)**	**1.23 (1.15–1.31)**	**2.63 (2.46–2.80)**	**3.64 (3.41–3.88)**	**2.44 (2.30–2.58)**
Visceral leishmaniasis	**1.15 (1.02–1.29)**	0.99 (0.90–1.09)	1.11 (1.00–1.23)	**1.63 (1.46–1.82)**	**3.26 (3.03–3.50)**	**2.75 (2.31–3.27)**
Schistosomiasis	**1.44 (1.09–1.91)**	1.07 (0.96–1.20)	**1.23 (1.18–1.29)**	**1.52 (1.43–1.61)**	**1.49 (1.36–1.63)**	**1.58 (1.42–1.77)**
Pulmonary tuberculosis	1.39 (0.97–2.00)*	1.15 (0.87–1.53)	0.92 (0.67–1.24)	**1.14 (1.04–1.24)**	**1.91 (1.82–2.01)**	**2.98 (2.62–3.38)**
Lepromatous leprosy	NE#	0.98 (0.46–2.08)†	**1.50 (1.09–2.05)**	**2.18 (1.95–2.44)**	**2.94 (2.84–3.05)**	**3.11 (2.84–3.40)**
Tuberculoid leprosy	NE	0.97 (0.65–1.46)	0.95 (0.85–1.06)	**0.85 (0.80–0.91)**	**0.82 (0.80–0.84)**	0.99 (0.93–1.05)
Typhoid fever	NE	0.80 (0.56–1.14)	0.77 (0.53–1.10)	**1.71 (1.06–2.77)**	1.05 (0.78–1.41)	0.90 (0.58–1.39)
Leptospirosis	**3.87 (2.26–6.60)**	1.08 (0.62–1.91)	**2.19 (1.76–2.73)**	**3.86 (3.03–4.92)**	**4.20 (3.27–5.39)**	**3.51 (2.43–5.07)**
Meningococcal meningitis	**1.26 (1.11–1.42)**	**1.24 (1.13–1.37)**	1.15 (0.94–1.39)	**1.19 (1.07–1.34)**	**1.39 (1.27–1.53)**	1.24 (0.80–1.93)
Hepatitis A	1.13 (0.99–1.30)	**1.10 (1.05–1.15)**	0.94 (0.89–1.00)	**1.25 (1.18–1.32)**	**1.39 (1.33–1.47)**	1.14 (0.95–1.37)
Severe dengue	0.95 (0.63**–**1.44)	**0.79 (0.68–0.93)**	0.93 (0.82–1.04)	1.03 (0.95–1.13)	**0.73 (0.62–0.86)**	1.08 (0.91–1.28)

Instances of significantly sex-biased incidence are highlighted in **bold** typeface; the age range for each age class is given in years.

* Significant male bias emerged when a few further cases were included in the calculations: IRR 1.47, 95%CI 1.12–1.94 (see text for details).

# NE, not estimated.

† Since only a few pediatric cases of lepromatous leprosy were recorded, for this analysis we added 1 to the number of cases and to the total population estimates for each year, and used also the cases reported in 2010.

### Visceral Leishmaniasis

Visceral leishmaniasis incidence is slightly but significantly male-biased during the first year of life, with a 15% higher risk among males; subsequently, risk becomes sex-unbiased in early and late childhood (Table 2). Male adolescents are at a 63% higher risk than females of developing visceral leishmaniasis. Across the reproductive age class, men are ∼3 times as likely as women to be diagnosed with the disease (Table 2). “Minipuberty” effects are observed only in the urban population; this result is not readily explicable, but the otherwise consistent results suggest that it might have arisen from noise in the 2006 records for this age class. IRRs are similar in rural and urban settings for the rest of age classes (Figure 3C). Exposure indices (serology and delayed-type hypersensitivity tests) again showed that contact with *Le. infantum* is sex-unbiased in Brazil (random-effects OR 0.96, 95%CI 0.79–1.15; data from 5866 individual tests; Figure 4) ([41]–[47] plus two reports [48], [49] presenting the same data) and elsewhere (e.g., [50]–[55]); the overall random-effects OR for these studies is 0.95 (95%CI 0.83–1.09; *N* = 14,971 tests).

### Manson’s Schistosomiasis

Schistosomiasis risk is overall male-biased during infancy (Figure 2, Table 2) yet the magnitude of the effect is small and it was not detected neither in the 2006 data (Text S2) nor when separately analyzing urban and rural populations from high-incidence states (Figure 3D). Male-female differences wane in early childhood and reappear among 5-9-year-olds, albeit the effect is again relatively small (a 23% increase in risk for males). Afterwards, male:female IRRs stabilize at ∼1.5, with 95%CI upper limits always <1.77 (Table 2). These patterns were fairly stable across years (Text S2), and reappeared in rural/urban comparisons, albeit male bias was larger in urban than in rural settings among adolescents and reproductive-age adults (Figure 3D). Serological evidence from apparently healthy Brazilian subjects shows that exposure to *Schistosoma mansoni* is male-biased among children [56], [57] and in the general population [58], with OR point estimates consistently above 2. The overall OR including all Latin American studies we retrieved (Brazil and Puerto Rico [56]–[60]) was 1.90 (95%CI 1.38–2.64; *N* = 5395 subjects; Figure 4). Brazilian prevalence surveys using the Kato-Katz method to detect *S. mansoni* eggs in stool samples from apparently healthy subjects [61]–[68] revealed male bias values similar to those seen in serological surveys; the random-effects OR was 2.12 (95%CI 1.42–3.18; *N* = 6891 subjects; Figure 4).

### Pulmonary Tuberculosis

During infancy, we observed an overall non-significant male bias (Table 2, Figure 2). The small number of cases among infants (122 notifications in 2006–2009) and the relatively large between-year variation (Text S2) need however be considered; for instance, the random-effects IRR reached significance (1.47, 95%CI 1.12–1.94) when the 90 cases reported in 2010 (retrieved November 7^th^ 2011) were included in the analysis, and male bias is even slightly larger if the three cases reported in 2006 are disregarded (IRR 1.50, 95%CI 1.13–1.98). These results suggest that there is a relatively small “minipuberty” effect in tuberculosis. Male bias shrinks during childhood and then rises progressively up to a peak IRR value of 2.98 in the population over 60 years of age (Table 2, Figure 2). Our meta-analysis of published Brazilian infection-without-disease tuberculosis surveys, including tuberculin skin tests (TST) [69]–[72] and, in one case, TST plus an IFN-γ release assay [73], revealed a marginally non-significant male bias in exposure to *Mycobacterium tuberculosis* (random-effects OR 1.16, 95%CI 1.00–1.36; *N* = 6725 subjects; Figure 4).

### Leprosy

Both adolescent and reproductive-age women are consistently more likely than men to be diagnosed with the milder, tuberculoid form of the disease; for the more severe lepromatous forms, the opposite pattern emerged, with males being at much higher risk, particularly after puberty and throughout the reproductive age class (Figure 2, Table 2). More specifically, average tuberculoid leprosy risk is even for both sexes in early and late childhood, although the precision of the estimate is low for younger children (Figure 2, Table 2). With the onset of puberty, girls become about 1.2 times as likely as boys to get a tuberculoid leprosy diagnosis; this female-biased pattern persists in the 20–59 age class, but disappears among the elderly (Table 2). During early childhood, the few reported cases of lepromatous leprosy (22 between 2006 and 2010) are not sex-biased (Table 2), paralleling the absence of any sex effect in tuberculoid disease. In late childhood, lepromatous leprosy risk shows a slight trend towards male bias; although 95%CIs included 1 in all year-specific analyses (Text S2), the random-effects mean IRR was estimated at 1.50 (95%CI 1.09–2.05) (Table 2). Male-biased lepromatous leprosy risk becomes evident during puberty and reproductive life, with males being about 2–3 times as likely to be diagnosed with this condition as females. In contrast with tuberculoid leprosy results, the risk of lepromatous forms remains markedly male-biased in the older population (Figure 2, Table 2). Exposure surveys conducted in Brazil [74]–[77] show that, even though results varied across studies, anti-*Mycobacterium leprae* seropositivity is overall sex-unbiased (random-effects OR 1.19, 95%CI 0.77–1.86; *N* = 12,444 subjects; Figure 4).

### Typhoid Fever

We did not observe any consistent male-female difference in our typhoid fever data (Figure 2, Table 2), but rather a somewhat erratic incidence pattern in which uncertainty was exacerbated by the relatively small number of records. Thus, the apparently male-biased risk in the 10–19 years old class (Table 2) emerges from inconsistent (and noticeably imprecise) year-specific estimates (Text S2). Out of 25 year- and age-specific comparisons, only in five was there significant male bias, whereas in 20 cases 95%CIs encompassed 1 (Text S2); this suggests that typhoid fever risk is overall sex-unbiased. We did not find any Brazilian study, but antibody surveys among apparently healthy adults conducted in Nigeria [78] and Vietnam [79] reveal no sex bias in exposure (random-effects OR 1.22, 95%CI 0.62–2.39; *N* = 3394 subjects; Figure 4).

### Leptospirosis

“Minipuberty” effects are overtly evident in leptospirosis, with similar male bias estimates in urban (IRR 2.00, 95%CI 1.20–3.34) and rural settings (IRR 2.04, 95%CI 1.67–2.50); subsequently, male-female differences disappear throughout childhood in both populations (Figure 3E). Male-biased risk soars with the onset of puberty to a peak IRR of 4.95 (95%CI 2.47–9.95) for rural youngsters aged 10–19, with a somewhat lower IRR for the same age class in urban environments. Reproductive-age rural men are at higher risk than women (IRR 4.22, 95%CI 3.09–5.76), with a much larger sex bias than that observed in urban settings (IRR 1.83, 95%CI 1.52–2.20). IRRs become again comparable in the older population (Figure 3E). For the economically active age classes (10–59 years old), the overall urban IRR is 1.98 (95%CI 1.75–2.25), *vs.* 4.33 (95%CI 3.49–5.38) in rural environments. To further explore the relative contributions of behavior and physiology to leptospirosis risk, we examined the proportion of males among 9498 incident cases (2006–2010 period) for which the most likely site of infection was traced to the working environment (91.7% males, 95%CI 91.2–92.1%; *N* = 3291 cases) or the home environment (69.6% males, 95%CI 69.0–70.2%; *N* = 6207 cases) (Figure 3F). Serological evidence of exposure to *Leptospira* spp. in Brazil [80]–[83] shows a moderately higher risk among males: a random-effects meta-analysis of four surveys (*N* = 3996 subjects) yielded an OR of 1.41 (95%CI 1.18–1.69) (Figure 4).

### Meningococcal Meningitis

Our data show a relatively small but significant male bias in infancy and among reproductive-age adults (risk ∼26% and ∼39% higher for males, respectively), whereas incidence is unbiased in late childhood and in people over 60 (Table 2, Figure 2). Meta-analyses of 17 published *Neisseria meningitidis* carrier surveys [84]–[100] showed a significant male bias in the general population (OR 1.52, 95%CI 1.28–1.81; *N* = 31,351 subjects) but a non-significant bias before adulthood (random-effects OR 1.16, 95%CI 0.97–1.38; *N* = 18,315 subjects) (Figure 4).

### Hepatitis A

The data reveal a small, non-significant male bias among infants, which reaches significance in early childhood; hepatitis A risk then becomes unbiased or slightly female-biased among 5–9 years old children (Table 2, Figure 2). Pubertal boys are at higher risk (∼25%) than girls; male bias peaks among reproductive-age adults (IRR 1.39), then wanes in older people, with all year-specific 95%CIs encompassing 1 except for 2007 (Text S2). Serological surveys conducted in Brazil [101]–[105] reveal sex-unbiased exposure patterns (random-effects OR 1.01, 95%CI 0.90–1.14; *N* = 8642 subjects; Figure 4). Except for a single Iranian study showing male-biased exposure [106], results from several further settings [107]–[109] reveal the same unbiased pattern (overall random-effects OR 1.01, 95%CI 0.90–1.13; *N* = 20,702 subjects).

### Severe Dengue Fever

Women between 20 and 59 years old are at a significantly higher risk (about 37%) than their male counterparts of developing severe forms of dengue (Table 2, Figure 2); this was consistent for all years analyzed except for 2009, when female bias was marginally non-significant (Text S2). In contrast, the overall female-biased pattern observed among 1-4-year-olds seen in Figure 2 results from year-specific data showing no significant sex bias except for 2007, when a slight female bias was observed (Text S2). Severe dengue fever risk is sex-unbiased for the rest of age class comparisons. A random-effects meta-analysis of the results of serological surveys conducted in Brazil [110]–[116] reveals that exposure to dengue virus is, on average, sex-unbiased (OR 0.96, 95%CI 0.80–1.14; *N* = 5920 subjects; Figure 4).

## Discussion

Using data from nearly 0.5 million individual cases or diagnostic test results, we conducted the first quantitative, comprehensive test of the predictions of the two main hypotheses formulated to explain sex-biased infectious disease risk in humans (see also ref. [3]). Three major findings seem to clearly contradict key BH predictions while closely matching those of the PH. First, male-biased infectious disease risk among infants cannot be accounted for by behavioral differences. Second, as predicted by the PH, risk is female-biased in tuberculoid leprosy but male-biased in lepromatous leprosy, and female-biased in severe dengue fever. And, third, exposure-prevalence indices are, with a few exceptions, sex-unbiased. The data, however, also suggest a role for gender-related behavior in diseases in which it is expected to affect exposure (e.g., schistosomiasis, leptospirosis, the leishmaniases) or result in contrasting risk factors for disease progression (e.g., smoking or alcohol use for tuberculosis among older adults).

Our findings are remarkably robust to inter-annual variation in official rates, to underreporting, and to the modeling procedure used to derive summary effect-size measures (see Text S2). Although our age categories are based on well-established physiological criteria [17], [117], we also tested whether an alternative definition of the ‘reproductive age class’, with two groups (20–39 and 40–59) instead of one, would qualitatively change our conclusions; the only difference was that no significant sex bias was found for typhoid fever risk (details not shown). Several study limitations must however be considered when interpreting our results. First, we used notification records of uncertain quality; still, we do not think it likely that quality issues may affect male and female data differentially: since we assess a relative measure (IRR), the results will hold if both are equally poor. Second, and as in other broad-scale appraisals, we do not have data on putative confounders; our strategy of (i) stratifying by age class, which allowed us to assess sex bias in infancy and childhood, and (ii) examining both disease-incidence and exposure-prevalence data alleviates, albeit does not eliminate, the problem of confounding. Third, although we analyzed data from a fairly diverse set of pathogens, these were not selected to test our hypotheses – they were given by data availability, by their public health importance, and by our ability to specify clear-cut predictions under each hypothesis. It is conceivable that a different set of diseases might lead to different conclusions; the relatively few studies addressing male-female differences in exposure and disease do suggest, however, that our findings are not confounded by taxonomy (see Text S2 for examples). Finally, we ignored pathogen and host variability and considered key physiological parameters only coarsely. For instance, our interpretation of age-stratified results relies on typical, average sex steroid levels for each age class [17]. More generally, while overall suggestive of support for the PH and partially at odds with the BH, our findings do not allow us to make direct causal claims. With these caveats in mind, we now proceed to discuss our results for each age class in relation to the key predictions of each major hypothesis.

Risk is male-biased during infancy for cutaneous and visceral leishmaniasis, schistosomiasis, leptospirosis, and meningococcal meningitis; in tuberculosis and hepatitis A, infant male bias is non-significant, and a similarly non-significant female bias is observed for severe dengue forms (Figure 2). The small number of incident cases precluded the assessment of typhoid fever and leprosy incidence during infancy. Since behavioral exposure to insect vectors (leishmaniases), contaminated water (schistosomiasis, leptospirosis), or meningococci-carrying respiratory droplets can be assumed equal for infants of both sexes, these results are clearly at odds with the BH.

In the case of pulmonary tuberculosis, we have seen (Results section) that male bias reaches significance when a few more cases are considered, and this could also be the case for hepatitis A (Table 2), suggesting that an adequately powered analysis is necessary to detect “minipuberty” effects in these diseases. Severe dengue fever is associated with a phenomenon known as antibody-dependent enhancement [29], [30]: upon secondary infection with a heterologous dengue virus serotype, cross-reacting antibodies developed during the primary infection event cannot neutralize the newly arrived virus, resulting in enhanced viral replication within monocytes and, consequently, more severe forms of the disease [29], [30]. Since infants are unlikely to get infected twice in their first year of life, both the PH and the BH predict a similar outcome (no bias) in this age class (Table 1).

What mechanisms may underlie these patterns of sex-biased infectious disease risk in infants? As mentioned in the Introduction, the PH predicts that the transient rise of sex steroid levels in the first year of life (“minipuberty” [17]) should yield the observed patterns. Other possibilities seem far less likely. For instance, genetic (chromosome) differences must apply roughly equally across age classes, and would not explain by themselves drastic changes in sex bias between infants and children. Differences in access to healthcare are another plausible explanation, but a study based on a country-representative sample of 110,000 Brazilian households revealed no gender-related difference during infancy and childhood [118]. It seems conceivable, however, that the reduction of infant mortality brought about by Cesarean delivery and neonatal intensive care units disproportionally benefited male infants, and particularly those with low weight and/or premature [119]. Since Cesarean delivery is particularly common in Brazil [120], we might speculate that this could have increased the pool of ‘frail’ male infants with a predisposition for infection. Yet, since the ‘male infant disadvantage’ these improvements helped reduce [119] is obviously not due to behavioral factors, we see this possibility as a further argument against the BH.

As expected for similar exposure and negligible sex steroid activity, bias either shrinks (cutaneous leishmaniasis, tuberculosis) or disappears (visceral leishmaniasis, schistosomiasis, leptospirosis) in children aged 1–4. Male bias is significant in meningococcal meningitis and hepatitis A, and female bias in severe dengue fever, among 1-4-year-olds. Male-biased risk emerges in late childhood for cutaneous leishmaniasis, schistosomiasis, lepromatous leprosy, leptospirosis, and, to a lesser extent, visceral leishmaniasis and meningococcal meningitis; a non-significant female bias is observed in typhoid fever, severe dengue fever, and hepatitis A, while risk is unbiased in tuberculosis and tuberculoid leprosy. Hence, the general pattern is one of even or slightly male-biased risk across childhood (Figure 2, Table 2). Since both the PH and the BH predict similar outcomes for this age group (Table 1), these results add little to our hypothesis-testing exercise. We also note that defining discrete age-classes may result in some confounding from cross-class effects in some individuals. For instance, physiological effects might be ‘carried over’ from infancy into early childhood, and puberty effects, physiological and behavioral, may already be apparent in older children.

Male-female differences are overtly seen during puberty; yet, their magnitude is nearly always smaller than across the reproductive period (Figure 2), mirroring average sex steroid levels in those age classes [17]. This is observed in cutaneous and visceral leishmaniases, tuberculosis, lepromatous leprosy, hepatitis A, and severe dengue fever; the difference is smaller in tuberculoid leprosy and meningococcal meningitis, and absent in schistosomiasis, leptospirosis, and typhoid fever (Figure 2). Reproductive-age men endure higher disease risk than women for intracellular pathogens, including protozoa (cutaneous and visceral leishmaniases), bacteria (tuberculosis, lepromatous leprosy), and viruses (hepatitis A), as well as for schistosomiasis and extracellular bacteria (leptospirosis and meningococcal meningitis). Particularly striking are the patterns in leprosy, a polymorphic disease whose clinical progression is largely driven by host immunity [121], [122]. Under the PH, different forms of leprosy are predicted to yield sharply contrasting sex bias patterns: while the milder tuberculoid form is linked to efficient Th1 responses, severe lepromatous forms are related to less protective, Th2-dominated responses (see [121], [122]). Since exposure to *M. leprae* is most likely sex-unbiased (cf. Figure 4), and disease progression behavior-independent, the BH predicts unbiased risk of leprosy, whereas the PH predicts tuberculoid forms to be female-biased and lepromatous forms to be male-biased (Table 1); this is precisely what we observed (Figure 2). The PH also predicts that severe dengue fever, linked to antibody-dependent enhancement [29], [30], should be female-biased in the reproductive-age population; our results also match this prediction (Figure 2). Among the elderly, male bias decreases visibly in cutaneous and visceral leishmaniasis, leptospirosis, meningococcal meningitis, and hepatitis A, whereas no clear changes are apparent for schistosomiasis, lepromatous leprosy, and typhoid fever; as predicted by the PH, female bias disappears in tuberculoid leprosy and severe dengue fever (Figure 2). Strongly male-biased tuberculosis risk is likely to stem from male-biased risk factors, such as smoking and alcohol intake [27].

We note that the results discussed so far not only provide partial support for the PH: they are also at odds with several key predictions of the BH, especially regarding disease progression/severity (leprosy, severe dengue) and sex-biased risk in same-behavior age classes. To provide a test of the third BH prediction, we built sociological contrasts comparing incidence rates in rural and urban populations for diseases in which differential risk-exposure is likely. The age-stratified patterns of male-female differences are comparable in both settings, although effect-sizes often differ: somewhat in contrast with our predictions (see Methods), post-pubertal male bias is larger in urban than in rural areas for cutaneous leishmaniasis and schistosomiasis; for visceral leishmaniasis, estimates are similar (Figure 3C). This suggests that urban women may be more protected from cutaneous leishmaniasis vectors (associated with forest/cropland) than rural ones, but both are equally exposed to the peridomestic vectors of visceral leishmaniasis (see also Dataset S2). Incidence profiles also suggest that cutaneous and visceral leishmaniasis transmission dynamics differ sharply (Figure 1). These data indicate that behavior, probably related to agriculture, exacerbates male-biased cutaneous leishmaniasis risk in some populations. Similarly, urban women seem more protected than rural ones from schistosomiasis (see Dataset S2), further supporting a role for behavior in modulating male-female differences. Since pathogenic (i.e., persistent) Th1 responses are overall stronger in women [15], [16] but exposure is most likely male-biased, we predicted schistosomiasis incidence to be less male-biased than *Schistosoma mansoni*-exposure indices (Table 1; see also below). For leptospirosis, the larger post-pubertal male bias in rural settings is likely related to higher male exposure in the working environment (Figure 3F), again suggesting a link with agriculture.

Finally, we tested the sharply contrasting PH and BH predictions about male-female differences in exposure-prevalence. Random-effects meta-analyses of male-female differences in published exposure-prevalence surveys showed that, as predicted by the PH and contrary to BH predictions, average exposure to the pathogens causing leishmaniases, leprosy, typhoid fever, hepatitis A, dengue, and, marginally, tuberculosis is sex-unbiased (Figure 4). Male bias among *N. meningitidis* carriers most likely arises from male-biased risk factors and from reporting bias (see Figure 4 caption). In agreement with our previous analyses, exposure to *Schistosoma* and *Leptospira* is male-biased, again suggesting a role for gender-specific behavior. As predicted taking both behavior and physiology into account, exposure-prevalence male bias is larger (about 60–70%) than disease-incidence male bias in schistosomiasis.

### Conclusions

We have presented a comprehensive test of the two major competing hypotheses proposed to explain male-female differences in infectious disease risk. Our findings suggest that sex-related physiology plays a major role in this phenomenon, albeit gender-specific behavior probably modulates infection risk in some instances. These results have potentially important implications from various perspectives. From an academic viewpoint, they provide novel insight into the population-level consequences of the evolutionary balance between sex and immunity to infection, suggesting that, in our species, the fitness trade-offs involved in such balance often manifest themselves as clear male-female differences in morbidity [6], [8], [16], [18], [22]. From a more practical stance, our results warn against unthinking extrapolation of biomedical research results, including vaccine development, across ages and genders [3], [4], [16], [20], [21]. Our findings also highlight sex as a major disease risk determinant that should not be treated (as is often the case in epidemiology) as a mere confounder; this, in turn, calls for better reporting standards in the epidemiological literature: sex-*and*-age-stratified results should always be provided for publication. Finally, our results suggest that, by considering immune-endocrine interactions [1], [9], [11]–[17], [123]–[125], insight could be gained not only into the epidemiology, but also into the pathogenesis, management, and prognosis of many infectious diseases [3]. Although specific research is obviously required to explore this possibility, a recent *E. coli* O104:H4 outbreak [126] provides a hint of the potential predictive power of the PH. Intriguingly, adult women were more often (and more seriously) affected in this outbreak [126]; behavior, in the form of food preferences, was proposed as a likely explanation [127]. Under the PH, however, this ‘atypical’ epidemiological pattern [126], [127] is the one to be expected if immunity plays a key role in pathogenesis. The subsequent demonstration that IgG antibody depletion has therapeutic value in the most severe forms of the disease [128] suggests that this prediction may indeed prove accurate.

In conclusion, and from a broader perspective, our analyses show that the development of a truly coherent view of infectious disease epidemiology demands an inter-disciplinary approach drawing from the diverse fields of microbiology, immunology, endocrinology, evolutionary biology, and sociology. Further research is needed to see to what extent our conclusions hold and can be generalized, but a growing body of evidence suggests that physiological sex differences likely underlie gender inequality in many infectious diseases.

## Supporting Information

Text S1 **MOOSE checklist.**
(PDF)

Text S2 **Sensitivity analyses and examples involving pathogens not included in our study.** Includes Tables S1–S11 (sensitivity to analytical procedure) and Figure S1 (year-specific results for each disease, 2006–2010).
(PDF)

Dataset S1 **Raw data used for crude incidence and male:female incidence rate ratio calculations in the general population (** **Table 2** **and** **Figures 1** **and** **2** **of the main text).**
(XLS)

Dataset S2 **Raw data used for the male:female incidence rate ratio calculations in sociological (rural/urban) contrasts (** **Figure 3** **of the main text).**
(XLS)
