# Supplementary material for: Sex Bias in Infectious Disease Epidemiology: Patterns and Processes
Source: PLoS One. 2013 Apr 24;8(4):e62390. doi: 10.1371/journal.pone.0062390 (PMC3634762; doi:10.1371/journal.pone.0062390)
Supplement: Text S1 — MOOSE checklist. (PDF) [file pone.0062390.s001.pdf]

Sex bias in infectious disease epidemiology: patterns and processes

Felipe Guerra-Silveira and Fernando Abad-Franch

MOOSE Checklist

Note: Since our appraisal of the literature was only one aspect of our hypothesis-testing exercise (which mainly relies on official incidence records), we did not perform a systematic review. Our meta-analyses are based on a selection of published papers reporting results for males and females separately; we only excluded papers with incomplete data – i.e., when a 2x2 table with positive and negative results for each sex could not be built. No report with these characteristics was excluded after assessment of its results

	Reported on page	Comments
**Reporting of background should include**		
Problem definition	1,3	
Hypothesis statement	1,4-5	See also Table 1
Description of study outcomes	4-7	
Type of exposure or intervention used	4-7	Age-stratified sex differences
Type of study designs used	7	
Study population	4-7	
Qualifications of searchers (eg librarians and investigators)		FA-F: MD, MSc, DLSHTM, PhD; FG-S: medical student
Search strategy, including time period used in the synthesis and key words	7	NOTE: non-systematic searches
Effort to include all available studies, including contact with authors	NA	See p. 7
Databases and registries searched	7	
Search software used, name and version, including special features used (eg explosion)	NA	Used standard Internet searches
Use of hand searching (eg reference lists of obtained articles)	7	All references used are presented
List of citations located and those excluded, including justification	NA	Only included papers were cited
Method of addressing articles published in languages other than English	NA	Authors fluent in English, Spanish, Portuguese
Method of handling abstracts and unpublished	NA	Not used

studies		
Description of any contact with authors	NA	Not done
**Reporting of methods should include**		
Description of relevance or appropriateness of studies assembled for assessing the hypothesis to be tested	7	
Rationale for the selection and coding of data (eg sound clinical principles or convenience)	7	
Documentation of how data were classified and coded (eg multiple raters, blinding and interrater reliability)	7	We used the data exactly as reported
Assessment of confounding (eg comparability of cases and controls in studies where appropriate)	NA	Only sex-stratified prevalence data were of interest
Assessment of study quality, including blinding of quality assessors, stratification or regression on possible predictors of study results	7	We were interested in sex-stratified data
Assessment of heterogeneity	7-8	Random-effects models used
Description of statistical methods (eg complete description of fixed or random effects models, justification of whether the chosen models account for predictors of study results, dose-response models, or cumulative meta-analysis) in sufficient detail to be replicated	7-8	
Provision of appropriate tables and graphics	Fig. 4	
**Reporting of results should include**		
Graphic summarizing individual study estimates and overall estimate	NA	Only the summary OR for each disease is reported
Table giving descriptive information for each study included	NA	
Results of sensitivity testing (eg subgroup analysis)	Fig. 4	
Indication of statistical uncertainty of findings	Throughout	95%CIs provided
**Reporting of discussion should include**		
Quantitative assessment of bias (eg publication bias)	NA	Not a systematic review; see also Fig. 4 caption
Justification for exclusion (eg exclusion of non-English language citations)	7	We only excluded papers with incomplete data
Assessment of quality of included studies	NA	We did not make a formal assessment
**Reporting of conclusions should include**		

Consideration of alternative explanations for observed results	Throughout	We compare two main hypotheses, and discuss other possibilities
Generalization of the conclusions (eg appropriate for the data presented and within the domain of the literature review)	Discussion	
Guidelines for future research	Discussion	
Disclosure of funding source	'Funding' section in the submission system	
