# Supplementary material for: Sex Bias in Infectious Disease Epidemiology: Patterns and Processes
Source: PLoS One. 2013 Apr 24;8(4):e62390. doi: 10.1371/journal.pone.0062390 (PMC3634762; doi:10.1371/journal.pone.0062390)
Supplement: Text S2 — Sensitivity analyses and examples involving pathogens not included in our study. Includes Tables S1–S11 (sensitivity to analytical procedure) and Figure S1 (year-specific results for each disease, 2006–2010). (PDF) [file pone.0062390.s002.pdf]

Sex bias in infectious disease epidemiology: patterns and processes

Felipe Guerra-Silveira and Fernando Abad-Franch

Supporting Text S2

1. Sensitivity analyses

We check the robustness of our results by performing sensitivity analyses, including (i) consistence of patterns across years (2006-2009); (ii) sensitivity to partial data availability (by using the incomplete 2010 records retrieved up to May 2011); and (iii) sensitivity to the use of different modeling procedures to estimate summary effect size measures. Overall quantitative results are reported in Table 2 of the main text.

Consistency across years. Figure S1 (below) shows the results of individual-year analyses. The general pattern is clearly one of consistent results except where the small number of cases results in large confidence intervals and, in some cases, suspect point estimates. As expected, year-specific estimates lack the precision of random-effects summary measures, and, for a few years and age classes, estimates are obviously unreliable (notably, leptospirosis among 1-4-year-olds in 2008, and tuberculosis or severe dengue fever among infants in 2006). These are, however, exceptions to the rule of consistency, which includes the apparently erratic results for typhoid fever data. Further details on year-specific results are presented in the Results section of the main text.

Incomplete data. Within each age class presented in Figure S1, the last estimate corresponds to the year 2010. By May 2011, which is when we retrieved the

compulsory-notification records used in our analyses, the 2010 data were labeled as "subject to revision" in the SINAN system (Brazilian Ministry of Health). For instance, 70 infant tuberculosis cases were recorded for 2010 by May 2011, but the figure reached 90 cases by November 2011. Therefore, our analysis of 2010 records up to May 2011 provides us with an indication of whether our results are robust to underreporting. Albeit with (as expected) somewhat wider confidence intervals (CIs), all 2010 estimates appear within the range of effect size estimates in previous years and overall for the 2006-2009 period.

Modeling procedure. We compare summary measures from year- and age-specific male:female incidence rate ratios (IRR) estimates and their 95% CIs as derived from three analytical procedures: (i) DerSimonian-Laird inverse-variance random-effects models [1] as implemented in Review Manager 5.1; this is the procedure we used to generate the results reported in the main text; (ii) the random-effects procedure described by Borenstein et al. [2]; this procedure uses year- and age class-specific IRR and 95% CI estimates to derive the summary effect measure, and is therefore sensitive to individual-year erroneous estimates (e.g., 2008 leptospirosis data for 1-4-year-olds, which result in a very large CI); and (iii) estimates of age-stratified cumulative incidence, i.e. the sum of cases divided by the population at the start of the study period (2006-2009); this procedure disregards between-year variation and may therefore estimate overly narrow CIs, but at the same time alleviates the effects of noisy year-specific data. The results of these analyses are presented in Tables S1 to S11 below; in those tables, "RevMan" indicates the first procedure, "Borenstein" the second, and "C-Incidence" the third. We highlighted in **bold** typeface the few instances in which statistical significance at $\alpha = 0.05$ varied with one particular procedure.

2. Examples involving pathogens not included in our study

As stated in the main text, we analyzed data from a fairly diverse set of pathogens (protozoa, one helminth, bacteria, and viruses) yet these were not selected purposefully to test our hypotheses – they were given by data availability, by their public health importance, and by our ability to specify clear-cut predictions under each major hypothesis. Hence, it is conceivable that a different set of diseases might lead to different conclusions. The relatively few studies addressing gender differences in exposure and disease do suggest, however, that our findings are not confounded by taxonomy. Examples of reports on pathogens we did not study include, but are not limited to, amebiasis (invasive disease male-biased, exposure unbiased) [3]; *Escherichia coli* O104:H4-associated hemolytic uremic syndrome (for which, given the likely involvement of IgG antibodies in pathogenesis [4], the physiological hypothesis predicts the observed higher risk for adult women [5]); infection following injury/surgery (male-biased) [6,7]; Q fever (disease male-biased, seroprevalence unbiased) [8]; measles (incidence male-biased, mortality female-biased) [9]; severe forms of H1N1 influenza (female-biased) [10]; and HIV1-related disease progression (faster in women) [11].

Supporting references

1. DerSimonian R, Laird N (1986) Meta–analysis in clinical trials. Control Clin Trials 7: 177–188.
2. Borenstein M, Hedges LV, Higgins JPT, Rothstein HR (2010) A basic introduction to fixed–effect and random–effects models for meta–analysis. Res Syn Meth 1: 97–111.
3. Acuna-Soto R, Maguire JH, Wirth DF (2000) Gender distribution in asymptomatic and invasive amebiasis. Am J Gastroenterol 95: 1277–1283.
4. Greinacher A, Friesecke S, Abel P, Dressel A, Stracke S, et al. (2011) Treatment of severe neurological deficits with IgG depletion through immunoadsorption in patients with *Escherichia coli* O104:H4-associated haemolytic uraemic syndrome: a prospective trial. Lancet 378: 1166–2273.

5. Frank C, Werber D, Cramer JP, Askar M, Faber M, et al., for the HUS investigation team (2011) Epidemic profile of Shiga–toxin–producing *Escherichia coli* O104:H4 outbreak in Germany. N Engl J Med 365: 1771–1780.
6. Crabtree TD, Pelletier SJ, Gleason TG, Pruett TL, Sawyer RG (1999) Gender-dependent differences in outcome after the treatment of infection in hospitalized patients. JAMA 282: 2143–2148.
7. Offner PJ, Moore EE, Biffl WL (1999) Male gender is a risk factor for major infections after surgery. Arch Surg 134: 935–940.
8. Tissot Dupont H, Raoult D, Brouqui P, Janbon F, Peyramond D, et al. (1992) Epidemiologic features and clinical presentation of acute Q fever in hospitalized patients: 323 French cases. Am J Med 93: 427–434.
9. Garenne M (1994) Sex differences in measles mortality: a world review. Int J Epidemiol 23: 632–642.
10. Klein SL, Passaretti C, Anker M, Olukoya P, Pekosz A (2010) The impact of sex, gender and pregnancy on 2009 H1N1 disease. Biol Sex Differ 1: 1–12.
11. Meier A, Chang JJ, Chan ES, Pollard RB, Sidhu HK, et al. (2009) Sex differences in the Toll–like receptor–mediated response of plasmacytoid dendritic cells to HIV-1. Nat Med 15: 955–959.

Supporting Tables

Table S1. Cutaneous leishmaniasis: sensitivity to modeling procedure

Age class (years)	Procedure	Mean IRR	95%CI low	95%CI up
<1	RevMan	2.24	1.95	2.57
	Borenstein	2.29	1.95	2.62
	C-Incidence	2.25	1.96	2.58
1-4	RevMan	1.16	1.06	1.27
	Borenstein	1.16	1.06	1.26
	C-Incidence	1.16	1.06	1.26
5-9	RevMan	1.23	1.15	1.31
	Borenstein	1.23	1.16	1.29
	C-Incidence	1.23	1.15	1.31
10-19	RevMan	2.63	2.46	2.80
	Borenstein	2.63	2.46	2.80
	C-Incidence	2.63	2.53	2.73
20-59	RevMan	3.64	3.41	3.88
	Borenstein	3.65	3.42	3.87
	C-Incidence	3.64	3.56	3.71
60+	RevMan	2.44	2.30	2.58
	Borenstein	2.44	2.30	2.58
	C-Incidence	2.42	2.31	2.55

Table S2. Visceral leishmaniasis: sensitivity to modeling procedure

Age class (years)	Procedure	Mean IRR	95%CI low	95%CI up
<1	RevMan	1.15	1.02	1.29
	Borenstein	1.16	1.03	1.28
	C-Incidence	1.15	1.03	1.29
1-4	RevMan	0.99	0.90	1.09
	Borenstein	0.99	0.90	1.09
	C-Incidence	0.99	0.93	1.06
5-9	RevMan	1.11	1.00	1.23
	Borenstein	1.12	**1.02**	1.22
	C-Incidence	1.11	1.00	1.23
10-19	RevMan	1.63	1.46	1.82
	Borenstein	1.63	1.52	1.74
	C-Incidence	1.63	1.46	1.83
20-59	RevMan	3.26	3.03	3.50
	Borenstein	3.26	3.16	3.36
	C-Incidence	3.26	3.03	3.51
60+	RevMan	2.75	2.31	3.27
	Borenstein	2.75	2.57	2.94
	C-Incidence	2.74	2.30	3.26

Table S3. Schistosomiasis: sensitivity to modeling procedure

Age class (years)	Procedure	Mean IRR	95%CI low	95%CI up
<1	RevMan	1.44	1.09	1.91
	Borenstein	1.50	1.08	1.92
	C-Incidence	1.35	1.18	1.54
1-4	RevMan	1.07	0.96	1.20
	Borenstein	1.07	0.96	1.18
	C-Incidence	1.07	0.96	1.20
5-9	RevMan	1.23	1.18	1.29
	Borenstein	1.23	1.19	1.28
	C-Incidence	1.23	1.18	1.29
10-19	RevMan	1.52	1.43	1.61
	Borenstein	1.52	1.44	1.61
	C-Incidence	1.49	1.45	1.52
20-59	RevMan	1.49	1.36	1.63
	Borenstein	1.50	1.36	1.64
	C-Incidence	1.45	1.43	1.47
60+	RevMan	1.58	1.42	1.77
	Borenstein	1.60	1.42	1.78
	C-Incidence	1.55	1.48	1.63

Table S4. Community-acquired pulmonary tuberculosis: sensitivity to modeling procedure

Age class (years)	Procedure	Mean IRR	95%CI low	95%CI up
<1	RevMan	1.39	0.97	2.00
	Borenstein	1.43	**1.07**	1.79
	C-Incidence	1.39	0.97	2.00
1-4	RevMan	1.15	0.87	1.53
	Borenstein	1.19	0.90	1.47
	C-Incidence	1.16	0.89	1.52
5-9	RevMan	0.92	0.67	1.24
	Borenstein	0.92	0.62	1.23
	C-Incidence	0.91	0.67	1.24
10-19	RevMan	1.14	1.04	1.24
	Borenstein	1.14	1.05	1.24
	C-Incidence	1.13	1.05	1.21
20-59	RevMan	1.91	1.82	2.01
	Borenstein	1.91	1.82	2.01
	C-Incidence	1.92	1.88	1.97
60+	RevMan	2.98	2.62	3.38
	Borenstein	3.06	2.69	3.43
	C-Incidence	2.84	2.67	3.03

Table S5. Lepromatous leprosy: sensitivity to modeling procedure

Age class (years)	Procedure	Mean IRR	95%CI low	95%CI up
<1	RevMan	-	-	-
	Borenstein	-	-	-
	C-Incidence	-	-	-
1-4	RevMan	1.20	0.50	2.89
	Borenstein	1.39	0.51	2.27
	C-Incidence	1.29	0.45	3.73
5-9	RevMan	1.50	1.09	2.05
	Borenstein	1.53	1.20	1.87
	C-Incidence	1.51	1.10	2.07
10-19	RevMan	2.18	1.95	2.44
	Borenstein	2.19	2.06	2.31
	C-Incidence	2.19	1.96	2.44
20-59	RevMan	2.94	2.84	3.05
	Borenstein	2.94	2.84	3.05
	C-Incidence	2.94	2.84	3.04
60+	RevMan	3.11	2.84	3.40
	Borenstein	3.12	2.85	3.40
	C-Incidence	3.09	2.92	3.27

Table S6. Tuberculoid leprosy: sensitivity to modeling procedure

Age class (years)	Procedure	Mean IRR	95%CI low	95%CI up
<1	RevMan	-	-	-
	Borenstein	-	-	-
	C-Incidence	-	-	-
1-4	RevMan	0.97	0.65	1.46
	Borenstein	1.03	0.58	1.48
	C-Incidence	1.02	0.78	1.33
5-9	RevMan	0.95	0.85	1.06
	Borenstein	0.95	0.84	1.06
	C-Incidence	0.95	0.85	1.06
10-19	RevMan	0.85	0.80	0.91
	Borenstein	0.86	0.79	0.92
	C-Incidence	0.85	0.80	0.91
20-59	RevMan	0.82	0.80	0.84
	Borenstein	0.82	0.80	0.85
	C-Incidence	0.82	0.80	0.84
60+	RevMan	0.99	0.93	1.05
	Borenstein	0.99	0.93	1.06
	C-Incidence	0.98	0.93	1.04

Table S7. Typhoid fever: sensitivity to modeling procedure

Age class (years)	Procedure	Mean IRR	95%CI low	95%CI up
<1	RevMan	-	-	-
	Borenstein	-	-	-
	C-Incidence	-	-	-
1-4	RevMan	0.80	0.56	1.14
	Borenstein	0.84	0.49	1.18
	C-Incidence	0.80	0.56	1.12
5-9	RevMan	0.77	0.53	1.10
	Borenstein	0.81	0.49	1.13
	C-Incidence	0.76	0.57	1.02
10-19	RevMan	1.71	1.06	2.77
	Borenstein	1.89	1.17	2.61
	C-Incidence	1.68	1.31	2.15
20-59	RevMan	1.05	0.78	1.41
	Borenstein	1.09	0.77	1.42
	C-Incidence	1.00	0.86	1.17
60+	RevMan	0.90	0.58	1.39
	Borenstein	0.93	0.50	1.36
	C-Incidence	0.89	0.58	1.37

Table S8. Leptospirosis: sensitivity to modeling procedure

Age class (years)	Procedure	Mean IRR	95%CI low	95%CI up
<1	RevMan	3.87	2.26	6.60
	Borenstein	4.51	2.67	6.34
	C-Incidence	4.04	2.38	6.86
1-4	RevMan	1.08	0.62	1.91
	Borenstein	2.37	0.46	4.28
	C-Incidence	1.13	0.73	1.77
5-9	RevMan	2.19	1.76	2.73
	Borenstein	2.33	1.83	2.83
	C-Incidence	2.21	1.78	2.75
10-19	RevMan	3.86	3.03	4.92
	Borenstein	3.98	3.00	4.95
	C-Incidence	3.77	3.41	4.17
20-59	RevMan	4.20	3.27	5.39
	Borenstein	4.29	3.27	5.32
	C-Incidence	4.11	3.91	4.33
60+	RevMan	3.51	2.43	5.07
	Borenstein	3.82	2.28	5.37
	C-Incidence	3.40	2.91	3.98

Table S9. Meningococcal meningitis: sensitivity to modeling procedure

Age class (years)	Procedure	Mean IRR	95%CI low	95%CI up
<1	RevMan	1.26	1.11	1.42
	Borenstein	1.26	1.14	1.38
	C-Incidence	1.26	1.11	1.42
1-4	RevMan	1.24	1.13	1.37
	Borenstein	1.24	1.15	1.34
	C-Incidence	1.24	1.13	1.37
5-9	RevMan	1.15	0.94	1.39
	Borenstein	1.16	0.93	1.40
	C-Incidence	1.14	**1.02**	1.28
10-19	RevMan	1.19	1.07	1.34
	Borenstein	1.20	1.06	1.33
	C-Incidence	1.20	1.08	1.32
20-59	RevMan	1.39	1.27	1.53
	Borenstein	1.40	1.28	1.51
	C-Incidence	1.39	1.27	1.53
60+	RevMan	1.24	0.80	1.93
	Borenstein	1.33	0.76	1.90
	C-Incidence	1.25	0.93	1.69

Table S10. Hepatitis A: sensitivity to modeling procedure

Age class (years)	Procedure	Mean IRR	95%CI low	95%CI up
<1	RevMan	1.13	0.99	1.30
	Borenstein	1.13	**1.00**	1.27
	C-Incidence	1.13	0.99	1.30
1-4	RevMan	1.10	1.05	1.15
	Borenstein	1.10	1.05	1.14
	C-Incidence	1.10	1.05	1.15
5-9	RevMan	0.94	0.89	**1.00**
	Borenstein	0.94	0.89	0.99
	C-Incidence	0.94	0.91	0.97
10-19	RevMan	1.25	1.18	1.32
	Borenstein	1.25	1.18	1.32
	C-Incidence	1.25	1.21	1.29
20-59	RevMan	1.39	1.33	1.47
	Borenstein	1.40	1.32	1.47
	C-Incidence	1.39	1.33	1.46
60+	RevMan	1.14	0.95	1.37
	Borenstein	1.16	0.94	1.39
	C-Incidence	1.14	0.95	1.36

Table S11.Severe dengue fever: sensitivity to modeling procedure

Age class (years)	Procedure	Mean IRR	95%CI low	95%CI up
<1	RevMan	0.95	0.63	1.44
	Borenstein	1.03	0.59	1.46
	C-Incidence	0.90	0.71	1.15
1-4	RevMan	0.79	0.68	0.93
	Borenstein	0.80	0.64	0.96
	C-Incidence	0.79	0.68	0.93
5-9	RevMan	0.93	0.82	1.04
	Borenstein	0.92	0.82	1.03
	C-Incidence	0.91	0.83	**0.99**
10-19	RevMan	1.03	0.95	1.13
	Borenstein	1.03	0.95	1.12
	C-Incidence	1.03	0.95	1.13
20-59	RevMan	0.73	0.62	0.86
	Borenstein	0.74	0.62	0.87
	C-Incidence	0.76	0.71	0.81
60+	RevMan	1.08	0.91	1.28
	Borenstein	1.09	0.92	1.27
	C-Incidence	1.07	0.91	1.27

Supporting Figure

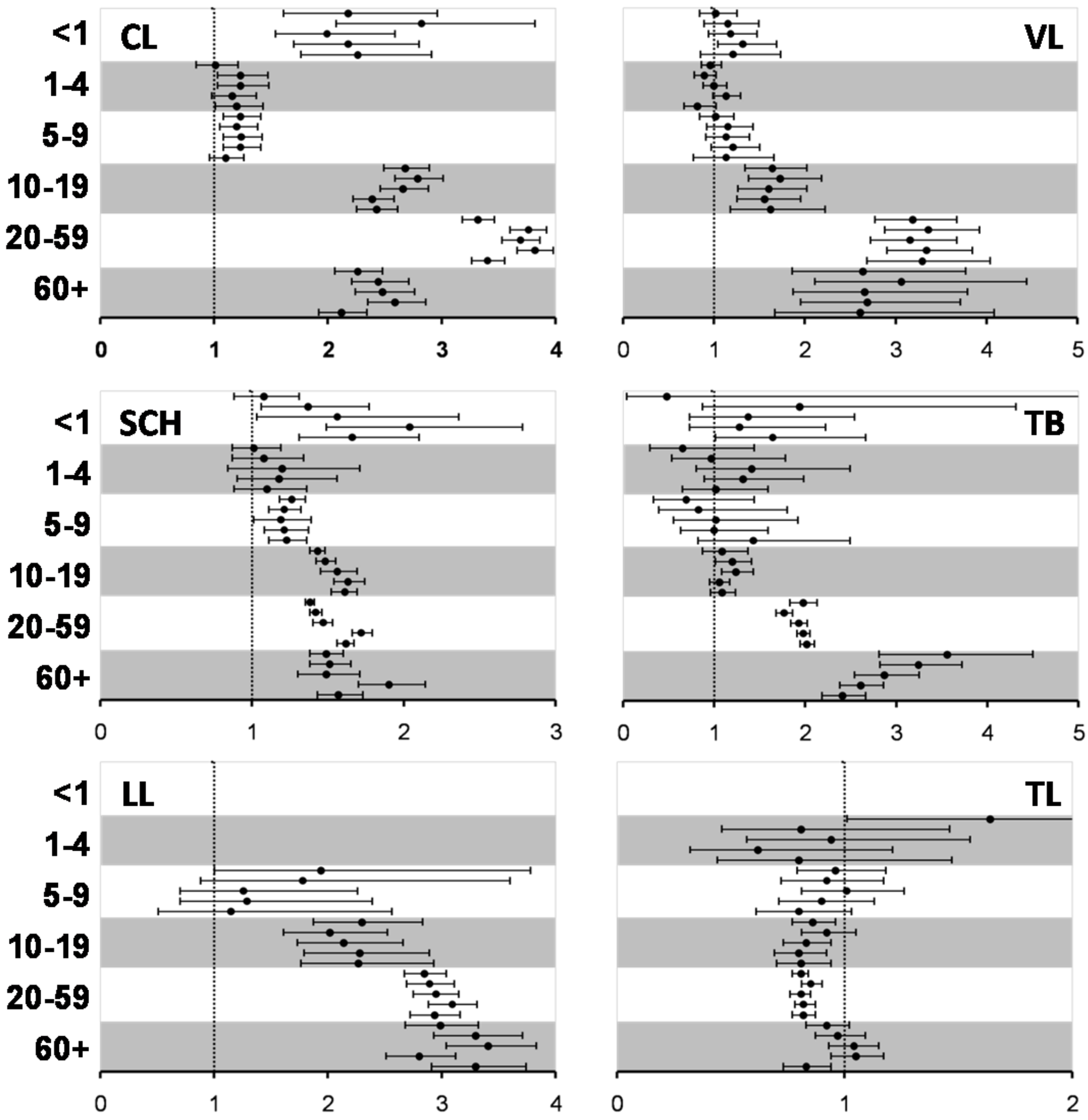

Figure S1. Sensitivity analyses: year- and age-specific incidence rate ratios and 95% confidence intervals for American cutaneous (CL) and visceral leishmaniasis (VL); schistosomiasis (SCH); pulmonary tuberculosis (TB); lepromatous leprosy (LL); and tuberculoid leprosy (TL); for each age class (gray/white bands), results for years 2006 (top) to 2010 (bottom) are presented

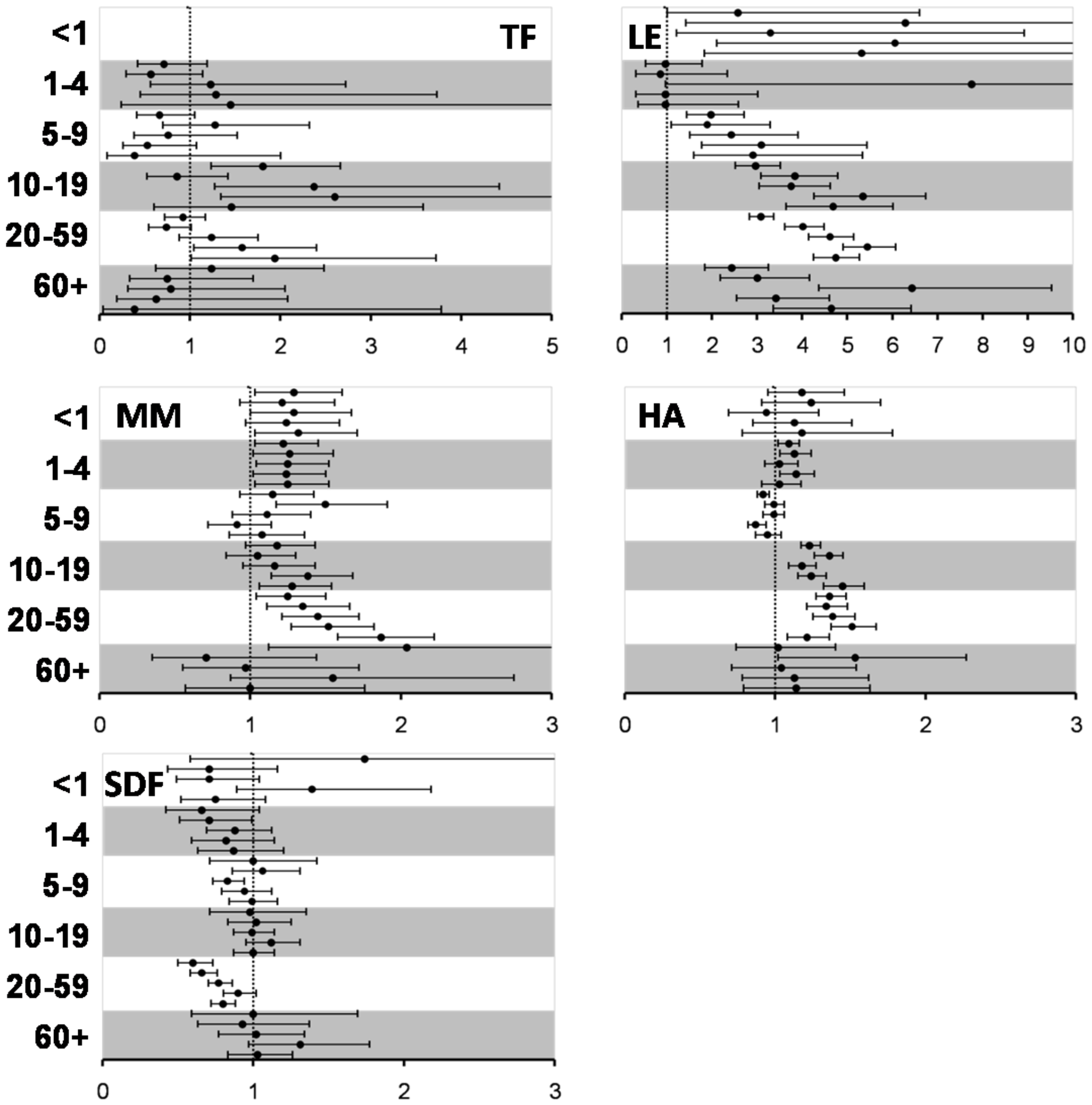

Figure S1 (continued)**.** Sensitivity analyses: year- and age-specific incidence rate ratios and 95% confidence intervals for typhoid fever (TF); leptospirosis (LE); meningococcal meningitis (MM); hepatitis A (HA); and severe dengue fever (SDF); for each age class (gray/white bands), results for years 2006 (top) to 2010 (bottom) are presented
